# Supplementary material for: An improved bacterial single-cell RNA-seq reveals biofilm heterogeneity
Source: eLife. 2024 Dec 17;13:RP97543. doi: 10.7554/eLife.97543 (PMC11651652; doi:10.7554/eLife.97543)
Supplement: Figure 1—source code 1. [file elife-97543-fig1-code1.zip › eLife-VOR-RA-2024-97543/Figure 1Source Code 1.docx]

**Figure 1B**

RiboD-PETRI data mRNA / total RNA Ratio

1.Since the reads1 and reads2 files contain different information, we cut the reads1 and reads2 files separately in order to improve the efficiency of data utilisation. Firstly, we removed the barcodes sequence contained in the front, and the last ten bases to remove the interference, and kept only 20 bases for mapping. The codes used in python were:

$zcat 0225-6_R1.fq.gz | seqkit grep -s -i -p ACACGACGCTCTTCCGA > r1.bc3.fastq

$cat r1.bc3.fastq | seqkit grep -s -i -p GGTCCTTGGCTTCGC > r1.bc32.fastq

$cat r1.bc32.fastq | seqkit grep -s -i -p CCTCCTACGCCAGA > r1.bc321.fastq

$zcat 0225-6_R2.fq.gz | seqkit grep -s -i -p ACACGACGCTCTTCCGA > r2.bc3.fastq

$cat r2.bc3.fastq | seqkit grep -s -i -p GGTCCTTGGCTTCGC > r2.bc32.fastq

$cat r2.bc32.fastq | seqkit grep -s -i -p CCTCCTACGCCAGA > r2.bc321.fastq

$cutadapt -u 120 -o r2.tirmed.fastq r2.bc321.fastq

$cutadapt -u -10 -o r2.tirmed2.fastq r2.tirmed.fastq

$cutadapt -u 120 -o r1.tirmed.fastq r1.bc321.fastq

$cutadapt -u -10 -o r1.tirmed2.fastq r1.tirmed.fastq

2. We then mapped the reads1 data and reads2 data of different species to the corresponding reference genomes. Then we can get the mapping result of reads1 and reads2 data for the following mRNA / total RNA reads ratio analysis.

For E.coli MG1655 K12:

$bwa aln Escherichia_coli_str_k_12_substr_mg1655.ASM584v2.dna.toplevel.fa.gz {sample}.R1.fq > {sample}.r1.sai

$bwa samse -f {sample}.r1.sam Escherichia_coli_str_k_12_substr_mg1655.ASM584v2.dna.toplevel.fa.gz {sample}.r1.sai {sample}.R1.fq

$samtools_0.1.18 view -Sb {sample}.r1.sam > {sample}.r1.bam

$featureCounts -T 6 -t transcript -g gene_id -a Escherichia_coli_str_k_12_substr_mg1655.ASM584v2.46.gtf -o {sample}.r1.txt {sample}.r1.bam

$bwa aln Escherichia_coli_str_k_12_substr_mg1655.ASM584v2.dna.toplevel.fa.gz RNA-seq/{sample}.R2.fq > {sample}.r2.sai

$bwa samse -f {sample}.r2.sam Escherichia_coli_str_k_12_substr_mg1655.ASM584v2.dna.toplevel.fa.gz {sample}.r2.sai {sample}.R2.fq

$samtools_0.1.18 view -Sb {sample}.r2.sam > {sample}.r2.bam

$featureCounts -T 6 -t transcript -g gene_id -a Escherichia_coli_str_k_12_substr_mg1655.ASM584v2.46.gtf -o {sample}.r2.txt {sample}.r2.bam

For Staphylococcus aureus 25923:

$bwa aln SA_25923.fasta {sample}.R1.fq > {sample}.r1.sai

$bwa samse -f {sample}.r1.sam SA_25923.fasta {sample}.r1.sai {sample}.R1.fq

$samtools_0.1.18 view -Sb {sample}.r1.sam > {sample}.r1.bam

$featureCounts -T 6 -t transcript -g gene_id -a SA_25923.gtf -o {sample}.r1.txt {sample}.r1.bam

$bwa aln SA_25923.fasta {sample}.R2.fq > {sample}.r2.sai

$bwa samse -f {sample}.r2.sam SA_25923.fasta {sample}.r2.sai {sample}.R2.fq

$samtools_0.1.18 view -Sb {sample}.r2.sam > {sample}.r2.bam

$featureCounts -T 6 -t transcript -g gene_id -a SA_25923.gtf -o {sample}.r2.txt {sample}.r2.bam

For Caulobacter crescentus NA1000:

$bwa aln NA1000.fa.gz r1.tirmed2.fastq > 0129-3.bc123.r1.sai

$bwa samse -f 0129-3.bc123.r1.sam NA1000.fa.gz 0129-3.bc123.r1.sai r1.tirmed2.fastq

$samtools_0.1.18 view -Sb 0129-3.bc123.r1.sam > 0129-3.bc123.r1.bam

$featureCounts -T 6 -t exon -g gene_id -a NA1000.gtf -o 0129-3.bc123.r1.txt 0129-3.bc123.r1.bam

$bwa aln NA1000.fa.gz r2.tirmed2.fastq > 0129-3.bc123.r2.sai

$bwa samse -f 0129-3.bc123.r2.sam NA1000.fa.gz 0129-3.bc123.r2.sai r2.tirmed2.fastq

$samtools_0.1.18 view -Sb 0129-3.bc123.r2.sam > 0129-3.bc123.r2.bam

$featureCounts -T 6 -t exon -g gene_id -a /NA1000.gtf -o 0129-3.bc123.r2.txt 0129-3.bc123.r2.bam

1. According to the expression matrix, calculate the proportion of rRNA and non-rRNA in it.

**Figure 1C**

Codes of analysis RiboD-PETRI and PETRI-seq scRNA-seq sequencing data

1.Since both reads1 and reads2 files contain information about barcode and genome, in order to improve the data utilisation efficiency of reads1 and reads2 files, we pre-processed the reads1 and reads2 files respectively. We used codes to combine the reads1 and reads2 files of scRNA-seq clean data (such as: "{sample}_R1.fq.gz" and "{sample}_R2.fq.gz") to mention barcode sequences and genome sequences, respectively, and processed them into four fastq files for downstream splitting of barcode and comparison processes. Make sure all fastq files have names in the form: {sample name}_{S#}_L00{#}_R1_001.fastq.gz , {sample name}_{S#}_L00{#}_R2_001.fastq.gz , {sample name}B_{S#}_L00{#}_R1_001.fastq.gz and {sample name}B_{S#}_L00{#}_R2_001.fastq.gz.

The codes are:

$cutadapt -g NNNNNNNNAGAATACACGACGCTCTTCCGATCT -o r1.fastq {sample}_R1.fq.gz

$cutadapt -g NNNNNNNNAGAATACACGACGCTCTTCCGATCT -o r2.fastq {sample}_R2.fq.gz

$cat r1.fastq | seqkit subseq -r 1:85 > {sample}_S5_L001_R1_001.fastq

$cat r2.fastq | seqkit subseq -r 80:100 > {sample}_S5_L001_R2_001.fastq

$cat r2.fastq | seqkit subseq -r 1:85 > {sample}B_S5_L001_R1_001.fastq

$cat r1.fastq | seqkit subseq -r 80:100 > {sample}B_S5_L001_R2_001.fastq

$gzip {sample}_S5_L001_R1_001.fastq

$gzip {sample}_S5_L001_R2_001.fastq

$gzip {sample}B_S5_L001_R1_001.fastq

$gzip {sample}B_S5_L001_R2_001.fastq

2.Create a folder named demo and then in the demo folder create the folders with the sample names : {sample} and {sample}B. Put {sample}_S5_L001_R1_001.fastq and {sample}_S5_L001_R2_001.fastq in the folder named {sample}; Put {sample}B_S5_L001_R1_001.fastq and {sample}B_S5_L001_R2_001.fastq in the folder named {sample}B.

3.Then we performed single-cell sequencing data analyses according to the processes and scripts of previous articles "Prokaryotic single-cell RNA sequencing by in situ combinatorial indexing". The scripts folder was also been put in "scripts" folder which we made some modifications owing to differences in python versions. The data are all in one lane, so we removed the step of merging the lanes. Then in the "demo" folder, run the following codes:

$python [path]/scripts/sc_pipeline_11.py {sample}_S5 {n_lanes}

$python [path]/scripts/sc_pipeline_11.py {sample}B_S5 {n_lanes}

# “sample” is sample name and S number (eg first1000_S5)

# n_lanes is the number of sequencing lanes for analysis - if lanes are merged, then the single file should be names with suffix _L001_R1_001.fastq.gz and n_lanes set to 1. The script will count lanes from 1 to n_lanes so always start numbering from 1.

# sc_pipeline_11 runs fastqc, quality filter, and barcode demultiplexing

For example:

$python path_to/scripts/sc_pipeline_11.py 1214-7_S5 1

$python path_to/scripts/sc_pipeline_11.py 1214_7B_S5 1

4.Look at {sample}_bc1_ReadsPerBC.eps, {sample}_bc1_kneePlot.eps, {sample}B_bc1_ReadsPerBC.eps and {sample}B_bc1_kneePlot.eps to determine number of BCs to include in further analysis. In the following analysis step, we only need to generate statistical tables for UMI, genes and cells, so we remove the step of synthesising the matrix. This script generates two files named as ("{sample}_CDS_v11_threshold_0_filtered_mapped_UMIs.txt" and "{sample}B_CDS_v11_threshold_0_filtered_mapped_UMIs.txt").

The codes were:

SCRIPT: [path]/scripts/pipeline.sh {sample} {n_BCs} {fasta} {gtf} {custom_name}

# n_BCs is number of BCs to include in further analysis (typically 10000-80000)

# fasta is location and name of fasta for alignment

# gtf is location and name of gtf for feature calling - see example.gtf for example format (specifically, gene names should be indicated by 'name=')

# Custom name is a new name for the sample, corresponding to maybe the gtf used or other specific input of the pipeline. For example, we might analyze the same cells by CDS or by exon and would indicate that in the custom name. Custom name can be the same as sample name if desired.

# pipeline.sh includes a number of cleanup commands at the end. If interested in intermediate files, these can be easily commented out.

For example:

$sh ./scripts/pipeline.sh 1214_7 10000 Escherichia_coli_str_k_12_substr_mg1655.ASM584v2.dna.toplevel.fa.gz Escherichia_coli_str_k_12_substr_mg1655.ASM584v2.46.gtf 1214_7_CDS

$sh ./scripts/pipeline.sh 1214_7B 10000 Escherichia_coli_str_k_12_substr_mg1655.ASM584v2.dna.toplevel.fa.gz Escherichia_coli_str_k_12_substr_mg1655.ASM584v2.46.gtf 1214_7B_CDS

5.The files ("{sample}_CDS_v11_threshold_0_filtered_mapped_UMIs.txt" and "{sample}B_CDS_v11_threshold_0_filtered_mapped_UMIs.txt") obtained in step 4 are combined into a matrix containing UMI, gene and barcode information.

The codes were:

$cat {sample}_CDS_v11_threshold_0_filtered_mapped_UMIs.txt {sample}B_CDS_v11_threshold_0_filtered_mapped_UMIs.txt > {sample}.txt

$sed -i "s/{sample}B/{sample}/g" {sample}.txt

$sed -i "s/Chromosome://g" {sample}.txt

$awk 'BEGIN{ FS=" ";OFS="+" }{ print $1,$2,$3 }' {sample}.txt > {sample}.2.txt

$cat {sample}.2.txt | sort | uniq > {sample}.3.txt

$awk 'BEGIN{ FS="+";OFS=" " }{ print $1,$3 }' {sample}.3.txt > {sample}.4.txt

$awk 'BEGIN{ FS=" ";OFS="+" }{ print $1,$2 }' {sample}.4.txt > {sample}.5.txt

$cat {sample}.5.txt | sort | uniq -c > {sample}.6.txt

$awk 'BEGIN{ FS=" ";OFS="\t" }{ print $2,$3,$1 }' {sample}.6.txt > {sample}.7.txt

$awk 'BEGIN{ FS="+";OFS="\t" }{ print $1,$2,$3 }' {sample}.7.txt > {sample}.8.txt

$awk 'BEGIN{ FS="\t\t";OFS="\t" }{ print $1,$2,$3 }' {sample}.8.txt > {sample}.9.txt

$awk 'BEGIN{ FS="\t";OFS="+" }{ print $1,$2,$3 }' {sample}.9.txt > {sample}.10.txt

$sed -i '1i\cell+gene+count' {sample}.10.txt

$awk 'BEGIN{ FS="+";OFS="\t" }{ print $1,$2,$3 }' {sample}.10.txt > {sample}.11.txt

$awk 'BEGIN{ FS="\t";OFS="," }{ print $1,$2,$3 }' {sample}.11.txt > {sample}.12.csv

>>>python

>>>import pandas as pd

>>>import numpy as np

>>>table = pd.read_csv("{sample}.12.csv")

>>>table=pd.pivot_table(table,index=["cell"],columns=["gene"],values=["count"],fill_value=0)

>>>df = pd.DataFrame(table)

>>>df.to_csv('{sample}.csv')

1. Download "{sample}.csv", removed rows and columns that clearly not belong to cells and genes (such as "cell", "16" and so on.) , and count the number of UMI in each cell in the gene expression matrix, and select different numbers of cells to calculate the median number of UMI.

**Figure 1D, E**

1. Single-cell sequencing data were combined with the UMI counts of all cells. The Pearson correlation coefficient (r) of UMI counts per gene (log2 UMIs) among scRNA-seq data and the reads per gene (log2 reads) of bulk RNA-seq data was calculated for common genes, excluding those with zero counts in either library.
2. The Pearson correlation coefficient (r) of UMI counts per gene (log2 UMIs) among different RiboD-PETRI datas were calculated for common genes, excluding those with zero counts in either library.

The codes were :

>f1<-read.table("{sample}1 vs {sample}2.txt")

>f2<-f1 + 1

>f3<-log2(f2)

>plot(f3[,1],f3[,2],xlab='{sample}1',ylab='{sample}2',las=1,mgp=c(1.5,0.5,0),cex.axis=0.8,mai=c(1,1,1,1))

>cor.test(f1.1.1[,1],f1.1.1[,2],method = "pearson")
