# Supplementary material for: An improved bacterial single-cell RNA-seq reveals biofilm heterogeneity
Source: eLife. 2024 Dec 17;13:RP97543. doi: 10.7554/eLife.97543 (PMC11651652; doi:10.7554/eLife.97543)
Supplement: Figure 2—source code 2. [file elife-97543-fig2-code2.zip › eLife-VOR-RA-2024-97543/Figure 2figure supplement 1 and Figure 2figure supplement 3 and Figure 2figure supplement 4Source Code 1.docx]

**Figure 2-figure supplement 1A-C, G-I**

Codes of analysis CC and SA scRNA-seq sequencing data

1.Since both reads1 and reads2 files contain information about barcode and genome, in order to improve the data utilisation efficiency of reads1 and reads2 files, we pre-processed the reads1 and reads2 files respectively. We used codes to combine the reads1 and reads2 files of scRNA-seq clean data (such as: "{sample}_R1.fq.gz" and "{sample}_R2.fq.gz") to mention barcode sequences and genome sequences, respectively, and processed them into four fastq files for downstream splitting of barcode and comparison processes. Make sure all fastq files have names in the form: {sample name}_{S#}_L00{#}_R1_001.fastq.gz , {sample name}_{S#}_L00{#}_R2_001.fastq.gz , {sample name}B_{S#}_L00{#}_R1_001.fastq.gz and {sample name}B_{S#}_L00{#}_R2_001.fastq.gz.

The codes are:

$cutadapt -g NNNNNNNNAGAATACACGACGCTCTTCCGATCT -o r1.fastq {sample}_R1.fq.gz

$cutadapt -g NNNNNNNNAGAATACACGACGCTCTTCCGATCT -o r2.fastq {sample}_R2.fq.gz

$cat r1.fastq | seqkit subseq -r 1:85 > {sample}_S5_L001_R1_001.fastq

$cat r2.fastq | seqkit subseq -r 80:100 > {sample}_S5_L001_R2_001.fastq

$cat r2.fastq | seqkit subseq -r 1:85 > {sample}B_S5_L001_R1_001.fastq

$cat r1.fastq | seqkit subseq -r 80:100 > {sample}B_S5_L001_R2_001.fastq

$gzip {sample}_S5_L001_R1_001.fastq

$gzip {sample}_S5_L001_R2_001.fastq

$gzip {sample}B_S5_L001_R1_001.fastq

$gzip {sample}B_S5_L001_R2_001.fastq

2.Create a folder named demo and then in the demo folder create the folders with the sample names : {sample} and {sample}B. Put {sample}_S5_L001_R1_001.fastq and {sample}_S5_L001_R2_001.fastq in the folder named {sample}; Put {sample}B_S5_L001_R1_001.fastq and {sample}B_S5_L001_R2_001.fastq in the folder named {sample}B.

3.Then we performed single-cell sequencing data analyses according to the processes and scripts of previous articles "Prokaryotic single-cell RNA sequencing by in situ combinatorial indexing". The scripts folder was also been put in "scripts" folder which we made some modifications owing to differences in python versions. The data are all in one lane, so we removed the step of merging the lanes. Then in the "demo" folder, run the following codes:

$python [path]/scripts/sc_pipeline_11.py {sample}_S5 {n_lanes}

$python [path]/scripts/sc_pipeline_11.py {sample}B_S5 {n_lanes}

# “sample” is sample name and S number (eg first1000_S5)

# n_lanes is the number of sequencing lanes for analysis - if lanes are merged, then the single file should be names with suffix _L001_R1_001.fastq.gz and n_lanes set to 1. The script will count lanes from 1 to n_lanes so always start numbering from 1.

# sc_pipeline_11 runs fastqc, quality filter, and barcode demultiplexing

For example:

$python path_to/scripts/sc_pipeline_11.py 1214-7_S5 1

$python path_to/scripts/sc_pipeline_11.py 1214_7B_S5 1

4.Look at {sample}_bc1_ReadsPerBC.eps, {sample}_bc1_kneePlot.eps, {sample}B_bc1_ReadsPerBC.eps and {sample}B_bc1_kneePlot.eps to determine number of BCs to include in further analysis. In the following analysis step, we only need to generate statistical tables for UMI, genes and cells, so we remove the step of synthesising the matrix. This script generates two files named as ("{sample}_CDS_v11_threshold_0_filtered_mapped_UMIs.txt" and "{sample}B_CDS_v11_threshold_0_filtered_mapped_UMIs.txt").

The codes were:

SCRIPT: [path]/scripts/pipeline.sh {sample} {n_BCs} {fasta} {gtf} {custom_name}

# n_BCs is number of BCs to include in further analysis (typically 10000-80000)

# fasta is location and name of fasta for alignment

# gtf is location and name of gtf for feature calling - see example.gtf for example format (specifically, gene names should be indicated by 'name=')

# Custom name is a new name for the sample, corresponding to maybe the gtf used or other specific input of the pipeline. For example, we might analyze the same cells by CDS or by exon and would indicate that in the custom name. Custom name can be the same as sample name if desired.

# pipeline.sh includes a number of cleanup commands at the end. If interested in intermediate files, these can be easily commented out.

5.The files ("{sample}_CDS_v11_threshold_0_filtered_mapped_UMIs.txt" and "{sample}B_CDS_v11_threshold_0_filtered_mapped_UMIs.txt") obtained in step 4 are combined into a matrix containing UMI, gene and barcode information.

The codes were:

$cat {sample}_CDS_v11_threshold_0_filtered_mapped_UMIs.txt {sample}B_CDS_v11_threshold_0_filtered_mapped_UMIs.txt > {sample}.txt

$sed -i "s/{sample}B/{sample}/g" {sample}.txt

$sed -i "s/Chromosome://g" {sample}.txt

$awk 'BEGIN{ FS=" ";OFS="+" }{ print $1,$2,$3 }' {sample}.txt > {sample}.2.txt

$cat {sample}.2.txt | sort | uniq > {sample}.3.txt

$awk 'BEGIN{ FS="+";OFS=" " }{ print $1,$3 }' {sample}.3.txt > {sample}.4.txt

$awk 'BEGIN{ FS=" ";OFS="+" }{ print $1,$2 }' {sample}.4.txt > {sample}.5.txt

$cat {sample}.5.txt | sort | uniq -c > {sample}.6.txt

$awk 'BEGIN{ FS=" ";OFS="\t" }{ print $2,$3,$1 }' {sample}.6.txt > {sample}.7.txt

$awk 'BEGIN{ FS="+";OFS="\t" }{ print $1,$2,$3 }' {sample}.7.txt > {sample}.8.txt

$awk 'BEGIN{ FS="\t\t";OFS="\t" }{ print $1,$2,$3 }' {sample}.8.txt > {sample}.9.txt

$awk 'BEGIN{ FS="\t";OFS="+" }{ print $1,$2,$3 }' {sample}.9.txt > {sample}.10.txt

$sed -i '1i\cell+gene+count' {sample}.10.txt

$awk 'BEGIN{ FS="+";OFS="\t" }{ print $1,$2,$3 }' {sample}.10.txt > {sample}.11.txt

$awk 'BEGIN{ FS="\t";OFS="," }{ print $1,$2,$3 }' {sample}.11.txt > {sample}.12.csv

>>>python

>>>import pandas as pd

>>>import numpy as np

>>>table = pd.read_csv("{sample}.12.csv")

>>>table=pd.pivot_table(table,index=["cell"],columns=["gene"],values=["count"],fill_value=0)

>>>df = pd.DataFrame(table)

>>>df.to_csv('{sample}.csv')

1. Download "{sample}.csv", removed rows and columns that clearly not belong to cells and genes (such as "cell", "16" and so on.) , and count the number of UMI in each cell in the gene expression matrix, and select cells with a UMI count greater than 15 to calculate the median UMI count.
2. Based on the gene expression matrix obtained in the previous step, count the number of genes detected in each cell. For gene/cell analysis, we used the matrix {sample}.csv, removed rows and columns that clearly not belong to cells and genes (such as "cell", "16" and so on.), and convert it to "txt" format file ({sample}.UMI-cell.txt) to counted the number of genes in each cell using the following codes in R software:

>a <- read.table("{sample}.UMI-cell.txt")

>a1 <- a[-1,-1]

>a1[a1 != 0] <- 1

>write.table(a1,"{sample}.gene-cell.xlsx",sep="\t", quote=F, row.names=T,col.names = T)

1. Sort the cells based on the number of genes and UMIs counted in each cell, and take the top 1,000, 5,000, 8,000 or 10,000 cells to calculate the median number of UMIs or genes contained in these cells.

**Figure 2-figure supplement 1D-F and Figure 2-figure supplement 3**

For Single-cell RiboD-PETRI data of Staphylococcus aureus:

>SA.data <- read.table("F:/0609-7.txt", sep="\t")

>SA.data1 <- SA.data[-1,-1]

>colnames(SA.data1) <- SA.data[1,-1]

>rownames(SA.data1) <- SA.data[-1,1]

>SA2 <- CreateSeuratObject(counts = t(SA.data1), project = "3", min.cells = 1, min.features =1)

>counts <- GetAssayData(object = SA2, slot = "counts")

>nonzero <- counts > 0

>keep_genes <- Matrix::rowSums(nonzero) >= 30

>filtered_counts <- counts[keep_genes, ]

>SA2 <- CreateSeuratObject(filtered_counts, meta.data = SA2@meta.data)

>SA2 <- subset(SA2,subset = nFeature_RNA> 15 & nFeature_RNA < 1000)

>VlnPlot(SA2, features = c("nFeature_RNA", "nCount_RNA"), ncol = 2)

>SA3 <- NormalizeData(SA2, normalization.method = "LogNormalize", scale.factor = 1e4)

>SA4 <- FindVariableFeatures(SA3, selection.method = 'vst', nfeatures = 500)

>SA5 <- ScaleData(object = SA4)

>SA6 <- RunPCA(SA5,features = VariableFeatures(object = SA5))

>DimPlot(SA6, reduction = 'pca')

>DimHeatmap(SA6, dims = 1:9, cells = 500, balanced = TRUE)

>SA7 <- FindNeighbors(SA6, dims = 1:6)

>SA8 <- FindClusters(SA7, resolution = 0.4)

>SA <- RunUMAP(SA8, dims = 1:6)

>DimPlot(SA, reduction = 'umap', label = TRUE, pt.size = 0.5)

>FeaturePlot(SA,features = c("nCount_RNA"), pt.size = 0.85)

>SA.markers <- FindAllMarkers(SA, only.pos = TRUE, min.pct = 0.25, logfc.threshold = 0.25)

>FeaturePlot(SA, features = c("KQ76-08605","KQ76-06280","KQ76-05040","KQ76-10515","KQ76-10520","KQ76-00870",

"KQ76-03955","KQ76-03895","KQ76-04970","KQ76-13335","KQ76-00740","KQ76-11725"))

**Figure 2-figure supplement 1J-L and Figure 2-figure supplement 4**

For Single-cell RiboD-PETRI data of CC NA1000:

>CC.data <- read.table("F:/0129-3.txt", sep="\t")

>CC.data1 <- CC.data[-1,-1]

>colnames(CC.data1) <- CC.data[1,-1]

>rownames(CC.data1) <- CC.data[-1,1]

>CC2 <- CreateSeuratObject(counts = t(CC.data1), project = "3", min.cells = 1, min.features =1)

>counts <- GetAssayData(object = CC2, slot = "counts")

>nonzero <- counts > 0

>keep_genes <- Matrix::rowSums(nonzero) >= 30

>filtered_counts <- counts[keep_genes, ]

>CC2 <- CreateSeuratObject(filtered_counts, meta.data = CC2@meta.data)

>CC3 <- subset(CC2, subset = nFeature_RNA >200& nFeature_RNA < 5000)##cell level

>VlnPlot(CC3, features = c("nFeature_RNA", "nCount_RNA"), ncol = 2)

>CC4 <- NormalizeData(CC3, normalization.method = "LogNormalize", scale.factor = 1e4)

>CC5 <- FindVariableFeatures(CC4, selection.method = 'vst', nfeatures = 500)

>CC6 <- ScaleData(object = CC5)

>CC7 <- RunPCA(CC6,features = VariableFeatures(object = CC11))# npcs = 100,

>DimPlot(CC7, group.by=NULL,reduction = 'pca')

>CC8 <- FindNeighbors(CC7, dims = 1:6)

>CC9 <- FindClusters(CC8, resolution = 0.4)

>CC <- RunUMAP(CC9, dims = 1:6)

>DimPlot(CC, reduction = 'umap', label = TRUE, pt.size = 0.8)

>CC.markers <- FindAllMarkers(CC, only.pos = TRUE, min.pct = 0.25, logfc.threshold = 0.25)

CC.markers

>FeaturePlot(CC, features = c("CCNA-00671","CCNA-02395","CCNA-03761", "CCNA-02553","CCNA-03106","CCNA-00259","CCNA-02361","CCNA-01780","CCNA-00986", "CCNA-03402","CCNA-00996","CCNA-03119"),combine = TRUE)

>FeaturePlot(CC, features = c("nCount_RNA"), pt.size = 0.5)
