# Supplementary material for: An improved bacterial single-cell RNA-seq reveals biofilm heterogeneity
Source: eLife. 2024 Dec 17;13:RP97543. doi: 10.7554/eLife.97543 (PMC11651652; doi:10.7554/eLife.97543)
Supplement: Figure 3—source code 1. [file elife-97543-fig3-code1.zip › eLife-VOR-RA-2024-97543/Figure 3 and Figure 3figure supplement 1 and Figure 3figure supplement 2Source Code 1.docx]

**Figure 3A**

Codes of analysis RiboD-PETRI scRNA-seq sequencing data

1.Since both reads1 and reads2 files contain information about barcode and genome, in order to improve the data utilisation efficiency of reads1 and reads2 files, we pre-processed the reads1 and reads2 files respectively. We used codes to combine the reads1 and reads2 files of scRNA-seq clean data (such as: "{sample}_R1.fq.gz" and "{sample}_R2.fq.gz") to mention barcode sequences and genome sequences, respectively, and processed them into four fastq files for downstream splitting of barcode and comparison processes. Make sure all fastq files have names in the form: {sample name}_{S#}_L00{#}_R1_001.fastq.gz , {sample name}_{S#}_L00{#}_R2_001.fastq.gz , {sample name}B_{S#}_L00{#}_R1_001.fastq.gz and {sample name}B_{S#}_L00{#}_R2_001.fastq.gz.

The codes are:

$cutadapt -g NNNNNNNNAGAATACACGACGCTCTTCCGATCT -o r1.fastq {sample}_R1.fq.gz

$cutadapt -g NNNNNNNNAGAATACACGACGCTCTTCCGATCT -o r2.fastq {sample}_R2.fq.gz

$cat r1.fastq | seqkit subseq -r 1:85 > {sample}_S5_L001_R1_001.fastq

$cat r2.fastq | seqkit subseq -r 80:100 > {sample}_S5_L001_R2_001.fastq

$cat r2.fastq | seqkit subseq -r 1:85 > {sample}B_S5_L001_R1_001.fastq

$cat r1.fastq | seqkit subseq -r 80:100 > {sample}B_S5_L001_R2_001.fastq

$gzip {sample}_S5_L001_R1_001.fastq

$gzip {sample}_S5_L001_R2_001.fastq

$gzip {sample}B_S5_L001_R1_001.fastq

$gzip {sample}B_S5_L001_R2_001.fastq

2.Create a folder named demo and then in the demo folder create the folders with the sample names : {sample} and {sample}B. Put {sample}_S5_L001_R1_001.fastq and {sample}_S5_L001_R2_001.fastq in the folder named {sample}; Put {sample}B_S5_L001_R1_001.fastq and {sample}B_S5_L001_R2_001.fastq in the folder named {sample}B.

3.Then we performed single-cell sequencing data analyses according to the processes and scripts of previous articles "Prokaryotic single-cell RNA sequencing by in situ combinatorial indexing". The scripts folder was also been put in "scripts" folder which we made some modifications owing to differences in python versions. The data are all in one lane, so we removed the step of merging the lanes. Then in the "demo" folder, run the following codes:

$python [path]/scripts/sc_pipeline_11.py {sample}_S5 {n_lanes}

$python [path]/scripts/sc_pipeline_11.py {sample}B_S5 {n_lanes}

# “sample” is sample name and S number (eg first1000_S5)

# n_lanes is the number of sequencing lanes for analysis - if lanes are merged, then the single file should be names with suffix _L001_R1_001.fastq.gz and n_lanes set to 1. The script will count lanes from 1 to n_lanes so always start numbering from 1.

# sc_pipeline_11 runs fastqc, quality filter, and barcode demultiplexing

For example:

$python path_to/scripts/sc_pipeline_11.py 1214-7_S5 1

$python path_to/scripts/sc_pipeline_11.py 1214_7B_S5 1

4.Look at {sample}_bc1_ReadsPerBC.eps, {sample}_bc1_kneePlot.eps, {sample}B_bc1_ReadsPerBC.eps and {sample}B_bc1_kneePlot.eps to determine number of BCs to include in further analysis. In the following analysis step, we only need to generate statistical tables for UMI, genes and cells, so we remove the step of synthesising the matrix. This script generates two files named as ("{sample}_CDS_v11_threshold_0_filtered_mapped_UMIs.txt" and "{sample}B_CDS_v11_threshold_0_filtered_mapped_UMIs.txt").

The codes were:

SCRIPT: [path]/scripts/pipeline.sh {sample} {n_BCs} {fasta} {gtf} {custom_name}

# n_BCs is number of BCs to include in further analysis (typically 10000-80000)

# fasta is location and name of fasta for alignment

# gtf is location and name of gtf for feature calling - see example.gtf for example format (specifically, gene names should be indicated by 'name=')

# Custom name is a new name for the sample, corresponding to maybe the gtf used or other specific input of the pipeline. For example, we might analyze the same cells by CDS or by exon and would indicate that in the custom name. Custom name can be the same as sample name if desired.

# pipeline.sh includes a number of cleanup commands at the end. If interested in intermediate files, these can be easily commented out.

For example:

$sh ./scripts/pipeline.sh 1214_7 10000 Escherichia_coli_str_k_12_substr_mg1655.ASM584v2.dna.toplevel.fa.gz Escherichia_coli_str_k_12_substr_mg1655.ASM584v2.46.gtf 1214_7_CDS

$sh ./scripts/pipeline.sh 1214_7B 10000 Escherichia_coli_str_k_12_substr_mg1655.ASM584v2.dna.toplevel.fa.gz Escherichia_coli_str_k_12_substr_mg1655.ASM584v2.46.gtf 1214_7B_CDS

5.The files ("{sample}_CDS_v11_threshold_0_filtered_mapped_UMIs.txt" and "{sample}B_CDS_v11_threshold_0_filtered_mapped_UMIs.txt") obtained in step 4 are combined into a matrix containing UMI, gene and barcode information.

The codes were:

$cat {sample}_CDS_v11_threshold_0_filtered_mapped_UMIs.txt {sample}B_CDS_v11_threshold_0_filtered_mapped_UMIs.txt > {sample}.txt

$sed -i "s/{sample}B/{sample}/g" {sample}.txt

$sed -i "s/Chromosome://g" {sample}.txt

$awk 'BEGIN{ FS=" ";OFS="+" }{ print $1,$2,$3 }' {sample}.txt > {sample}.2.txt

$cat {sample}.2.txt | sort | uniq > {sample}.3.txt

$awk 'BEGIN{ FS="+";OFS=" " }{ print $1,$3 }' {sample}.3.txt > {sample}.4.txt

$awk 'BEGIN{ FS=" ";OFS="+" }{ print $1,$2 }' {sample}.4.txt > {sample}.5.txt

$cat {sample}.5.txt | sort | uniq -c > {sample}.6.txt

$awk 'BEGIN{ FS=" ";OFS="\t" }{ print $2,$3,$1 }' {sample}.6.txt > {sample}.7.txt

$awk 'BEGIN{ FS="+";OFS="\t" }{ print $1,$2,$3 }' {sample}.7.txt > {sample}.8.txt

$awk 'BEGIN{ FS="\t\t";OFS="\t" }{ print $1,$2,$3 }' {sample}.8.txt > {sample}.9.txt

$awk 'BEGIN{ FS="\t";OFS="+" }{ print $1,$2,$3 }' {sample}.9.txt > {sample}.10.txt

$sed -i '1i\cell+gene+count' {sample}.10.txt

$awk 'BEGIN{ FS="+";OFS="\t" }{ print $1,$2,$3 }' {sample}.10.txt > {sample}.11.txt

$awk 'BEGIN{ FS="\t";OFS="," }{ print $1,$2,$3 }' {sample}.11.txt > {sample}.12.csv

>>>python

>>>import pandas as pd

>>>import numpy as np

>>>table = pd.read_csv("{sample}.12.csv")

>>>table=pd.pivot_table(table,index=["cell"],columns=["gene"],values=["count"],fill_value=0)

>>>df = pd.DataFrame(table)

>>>df.to_csv('{sample}.csv')

1. Download "{sample}.csv", removed rows and columns that clearly not belong to cells and genes (such as "cell", "16" and so on.), and count the number of UMI in each cell in the gene expression matrix, and select cells with a UMI count greater than 15 to calculate the median UMI count.

**Figure 3B, C**

1. Based on the gene expression matrix obtained in the previous step, count the number of genes detected in each cell. For gene/cell analysis, we used the matrix {sample}.csv, removed rows and columns that clearly not belong to cells and genes (such as "cell", "16" and so on.), and convert it to "txt" format file ({sample}.UMI-cell.txt) to counted the number of genes in each cell using the following codes in R software:

>a <- read.table("{sample}.UMI-cell.txt")

>a1 <- a[-1,-1]

>a1[a1 != 0] <- 1

>write.table(a1,"{sample}.gene-cell.xlsx",sep="\t", quote=F, row.names=T,col.names = T)

1. Sort the cells based on the number of genes and UMIs counted in each cell, and take the top 1,621, 3,999 cells to calculate the median number of UMIs or genes contained in these cells.

**Figure 3D, E, H and Figure 3-figure supplement 1C-F, Figure 3-figure supplement 2**

For Single-cell RiboD-PETRI data of E .coli biofilm:

>library(SeuratObject)

>library(Seurat)

>library(patchwork)

>library(dplyr)

>library(harmony)

>Data1 <- read.table("F:/1214-7.txt", sep="\t")

>Data11 <- Data1[-1,-1]

>colnames(Data11) <- Data1[1,-1]

>rownames(Data11) <- Data1[-1,1]

>Data2 <- read.table("F:/1214-8.txt", sep="\t")

>Data21 <- Data2[-1,-1]

>colnames(Data21) <- Data2[1,-1]

>rownames(Data21) <- Data2[-1,1]

>ecoli24h_1 <- CreateSeuratObject(counts = t(Data11), project = "Data1", min.cells = 5)

>ecoli24h_1$stim <- "replicate1"

>ecoli24h_1_1 <- subset(ecoli24h_1, subset = nFeature_RNA > 100 & nFeature_RNA < 2000)

>ecoli24h_1_2 <- NormalizeData(ecoli24h_1_1, verbose = FALSE)

>ecoli24h_1_3 <- FindVariableFeatures(ecoli24h_1_2, selection.method = "vst", nfeatures = 500)

>ecoli24h_2 <- CreateSeuratObject(counts = t(Data21), project = "Data2", min.cells = 5)

>ecoli24h_2$stim <- "replicate2"

>ecoli24h_2_1 <- subset(ecoli24h_2, subset = nFeature_RNA >100 & nFeature_RNA < 2000)

>ecoli24h_2_2 <- NormalizeData(ecoli24h_2_1, verbose = FALSE)

>ecoli24h_2_3 <- FindVariableFeatures(ecoli24h_2_2, selection.method = "vst", nfeatures = 500)

>biofilm.anchors <- FindIntegrationAnchors(object.list = list(ecoli24h_1_3, ecoli24h_2_3), dims = 1:20)

>biofilm.combined <- IntegrateData(anchorset = biofilm.anchors, dims = 1:20)

>VlnPlot(biofilm.combined, features = c("nFeature_RNA", "nCount_RNA"), ncol = 2)

>DefaultAssay(biofilm.combined) <- 'integrated'

>biofilm.combined_1 <- ScaleData(biofilm.combined, verbose = FALSE)

>biofilm.combined_2 <- RunPCA(biofilm.combined_1, npcs = 30, verbose = FALSE)

>biofilm.combined_2 <- RunHarmony(biofilm.combined_2, group.by.vars = "orig.ident")

(*This step can be chosen to be done or not, corresponding to the results before or after correcting the batch effect, respectively*)

>DimPlot(biofilm.combined_2, group.by=NULL,reduction = 'pca')

>biofilm.combined_4 <- RunUMAP(biofilm.combined_2, reduction = 'pca', dims = 1:20)

>biofilm.combined_5 <- FindNeighbors(biofilm.combined_4, reduction = 'pca', dims = 1:20)

>biofilm.combined_6 <- FindClusters(biofilm.combined_5, resolution = 0.3)

>DimPlot(biofilm.combined_6, reduction = 'umap', group.by = 'stim')

>DimPlot(biofilm.combined_6, reduction = 'umap', label = TRUE)

>pbmc.markers <- FindAllMarkers(biofilm.combined_6, only.pos = TRUE, min.pct = 0.25, logfc.threshold = 0.2)

>FeaturePlot(biofilm.combined_6, features = c("b0833"), pt.size = 0.85)

>pbmc.markers %>% group_by(cluster) %>% top_n(n = 10, wt = avg_log2FC) -> top10

>DoHeatmap(biofilm.combined_6, features = top10$gene) + NoLegend()

>FeaturePlot(biofilm.combined_6,features = c("nCount_RNA"), pt.size = 0.85)

>FeaturePlot(biofilm.combined_6, features = c("b0389","b3256","b1451","b4067","b4071","b0967","b3454","b4374","b0041","b3089","b0833","b2919","b2954","b0641","b2446","b1845"), pt.size = 0.85)

>DotPlot(object = biofilm.combined_6,features =c("b2446","b0641","b2954","b0833","b3089","b0041","b4374","b0967","b1451","b3256","b0389"))

**Figure 3F**

>library("clusterProfiler")

>library(topGO)

>library(Rgraphviz)

>library(pathview)

>library(org.EcK12.eg.db)

>library(ggnewscale)

>Tn1<-read.table("24h GO pvalue 0.001.txt")

>Tn5<-Tn1[,1]

>ego <- enrichGO(

gene = Tn5,

keyType = "ENTREZID",

OrgDb = org.EcK12.eg.db,

ont = "CC",

pAdjustMethod = "BH",

pvalueCutoff = 0.4,

qvalueCutoff =0.4,

readable = TRUE)

>barplot(ego,color = "pvalue",showCategory = 2)

**Figure 3G**

>library(installr)

>library(CreateSeuratObject)

>library(Seurat)

>library(patchwork)

>library(SeuratObject)

>library(Matrix)

>Ecoli3h.data<-read.table("3h.txt")

>Ecoli3h.data1 <- Ecoli3h.data[-1,-1]

>colnames(Ecoli3h.data1) <- Ecoli3h.data[1,-1]

>rownames(Ecoli3h.data1) <- Ecoli3h.data[-1,1]

>Ecoli3h2 <- CreateSeuratObject(counts = t(Ecoli3h.data1), project = "3", min.cells = 1, min.features =1)

>Ecoli3h3 <- subset(Ecoli3h2, subset = nFeature_RNA >200 & nFeature_RNA < 5000)##cell level

>plot2 <- FeatureScatter(Ecoli3h3, feature1 = "nCount_RNA", feature2 = "nFeature_RNA")

>Ecoli3h4 <- NormalizeData(Ecoli3h3, normalization.method = "LogNormalize", scale.factor = 1e4)

>Ecoli3h5 <- FindVariableFeatures(Ecoli3h4, selection.method = 'vst', nfeatures = 500)

>Ecoli3h6 <- ScaleData(Ecoli3h5)

>Ecoli3h7 <- RunPCA(Ecoli3h6,features = VariableFeatures(object = Ecoli3h6))# npcs = 100,

>Ecoli3h10 <- FindNeighbors(Ecoli3h7, dims = 1:6)

>Ecoli3h11 <- FindClusters(Ecoli3h10, resolution = 0.3)

>Ecoli3h <- RunUMAP(Ecoli3h11, dims = 1:6)

>DimPlot(Ecoli3h, reduction = 'umap', label = TRUE, pt.size = 0.85)

>DotPlot(object = Ecoli3h,features =c("b2446","b0641","b2954","b0833","b3089","b0041","b4374","b0967","b1451","b3256","b0389"))

**Figure 3-figure supplement 1A**

1. For UMI/cell analysis, we used {sample}_CDS_v11_threshold_0_filtered_mapped_UMIs.txt and {sample}B_CDS_v11_threshold_0_filtered_mapped_UMIs.txt to counted the number of UMIs in each cell using the following codes:

$cat {sample}_CDS_v11_threshold_0_filtered_mapped_UMIs.txt {sample}B_CDS_v11_threshold_0_filtered_mapped_UMIs.txt > {sample}.txt

$sed -i "s/{sample}B/{sample}/g" {sample}.txt

$sed -i "s/Chromosome://g" {sample}.txt

$awk 'BEGIN{ FS=" ";OFS="+" }{ print $1,$2,$3}' {sample}.txt > {sample}.2.txt

$cat {sample}.2.txt | sort | uniq > {sample}.3.txt

$sed -i "/+VN+1.6/d" {sample}.3.txt

$sed -i "/+SN+Chro:LN/d" {sample}.3.txt

$sed -i "/+ID+bwa/d" {sample}.3.txt

$sed -i "/ambiguous/d" {sample}.3.txt

$awk 'BEGIN{ FS="+";OFS=" " }{ print $1}' {sample}.3.txt > {sample}.4.txt

$cat {sample}.4.txt | sort | uniq -c > {sample}.5.txt

$mv {sample}.5.txt {sample}.UMI-cell.txt

1. For reads/cell analysis, we used {sample}_CDS_v11_threshold_0_filtered_mapped_UMIs.txt and {sample}B_CDS_v11_threshold_0_filtered_mapped_UMIs.txt to counted the number of reads in each cell using the following codes:

$cat {sample}_CDS_v11_threshold_0_filtered_mapped_UMIs.txt {sample}B_CDS_v11_threshold_0_filtered_mapped_UMIs.txt > {sample}.txt

$sed -i "s/{sample}B/{sample}/g" {sample}.txt

$sed -i "s/Chromosome://g" {sample}.txt

$awk 'BEGIN{ FS=" ";OFS="+" }{ print $1,$2,$3,$4}' {sample}.txt > {sample}.2.txt

$sed -i "/+VN+1.6/d" {sample}.2.txt

$sed -i "/+SN+Chro:LN/d" {sample}.2.txt

$sed -i "/+ID+bwa/d" {sample}.2.txt

$sed -i "/ambiguous/d" {sample}.2.txt

$awk 'BEGIN{ FS="+";OFS=" " }{ print $1,$4}' {sample}.2.txt > {sample}.3.txt

$awk '{sum[$1]+=$2}END{for(i in sum)print i" "sum[i]}' {sample}.3.txt > {sample}.4.txt

$mv {sample}.4.txt {sample}.reads-cell.txt

1. Calculate the correlation coefficient between the UMI count and reads count of each counted cell.

The codes were :

>f1<-read.table("{sample}1 vs {sample}2.txt")

>f2<-f1 + 1

>f3<-log2(f2)

>plot(f3[,1],f3[,2],xlab='{sample}1',ylab='{sample}2',las=1,mgp=c(1.5,0.5,0),cex.axis=0.8,mai=c(1,1,1,1))

>cor.test(f1.1.1[,1],f1.1.1[,2],method = "pearson")

**Figure 3-figure supplement 1B**

1. We separately counted the number of UMIs corresponding to each gene in the gene expression matrices of two duplicate samples, and screened for genes with non-zero counts in both samples.
2. Then, we counted the correlation coefficients of the expression levels of the same gene in different samples.

The codes were:

>f1<-read.table("{sample}1 vs {sample}2.txt")

>f2<-f1 + 1

>f3<-log2(f2)

>plot(f3[,1],f3[,2],xlab='{sample}1',ylab='{sample}2',las=1,mgp=c(1.5,0.5,0),cex.axis=0.8,mai=c(1,1,1,1))

>cor.test(f1.1.1[,1],f1.1.1[,2],method = "pearson")
